# New Appliances and Things Medical

**Published:** 1907-03-23

**Authors:** 


### NEW APPLIANCES AND THINGS MEDICAL.

**[We shall be glad to receive, at our Office, 28 & 29 Southampton Street, Strand, London, W.C., from the manufacturers, specimens of all new preparations and appliances which may be brought out from time to time.]**

### HORLICK'S MALTED MILK.

(The Horlick's Food Company, 34 Farringdon Road, London, E.C.)

We have received a sample of this well-known preparation. It consists of 50 per cent. desiccated milk, 26.25 per cent. of wheaten flour, 23 per cent. barley malt, and 0.75 per cent. potassium and sodium bicarbonates. From this composition it follows that malted milk is an exceedingly nutritive preparation and is also very easily digested. The diastase present in the malted barley not only converts practically the whole of the starch into dextrine, or so-called soluble starch, but it also acts upon the proteid, that is the casein of the milk, and renders this incapable of being curdled in the stomach. These two features, along with the fact that by the addition of the wheaten flour and barley malt, both the proteid and carbo-hydrate contents are greatly increased, render the food of special value. The fat which is contained in the food is practically all derived from the milk. It must, however, be admitted that when the food is made up according to the directions with water, its fat content, as calculated from the analyses with which we have been provided, is below that of ordinary cow's or human milk. It is, however, quite simple to reinforce the fat by the addition of cream or by making the food with milk instead of water. Our experience with this food, from the clinical standpoint, is that patients like it and do well on it. We think that it actually possesses the merits ascribed to it, and it is therefore to be recommended.

